# Cardiovascular magnetic resonance feature tracking in pigs: a reproducibility and sample size calculation study

**DOI:** 10.1007/s10554-020-01767-y

**Published:** 2020-01-16

**Authors:** A. Faragli, R. Tanacli, C. Kolp, T. Lapinskas, C. Stehning, B. Schnackenburg, F. P. Lo Muzio, S. Perna, B. Pieske, E. Nagel, H. Post, S. Kelle, A. Alogna

**Affiliations:** 1 Department of Internal Medicine and Cardiology, Charité – Universitätsmedizin Berlin, Campus Virchow-Klinikum, Augustenburgerplatz 1, 13353 Berlin, Germany; 2 Berlin Institute of Health (BIH), Berlin, Germany; 3 DZHK (German Centre for Cardiovascular Research), partner site, Berlin, Germany; 4 Department of Internal Medicine / Cardiology, Deutsches Herzzentrum Berlin, Augustenburger Platz 1, 13353 Berlin, Germany; 5 Department of Cardiology, Medical Academy, Lithuanian University of Health Sciences, Eiveniu Street 2, 50161 Kaunas, Lithuania; 6 Clinical Science, Philips Healthcare, Röntgenstr. 24, 22335 Hamburg, Germany; 7 Department of Surgery, Dentistry, Paedriatics and Gynaecology, University of Verona, Via S. Francesco 22, 37129 Verona, Italy; 8 Department of Medicine and Surgery, University of Parma, Via Gramsci 14, 43126 Parma, Italy; 9 Department of Biology, College of Science, University of Bahrain, Sakhir Campus, P.O. Box 32038, Zallaq, Bahrain; 10 Institute of Experimental and Translational Cardiac Imaging, DZHK Centre for Cardiovascular Imaging, Goethe University Hospital Frankfurt, Theodor-Stern-Kai 7, 60590 Frankfurt, Germany; 11 Department of Cardiology, Contilia Heart and Vessel Centre, St. Marien-Hospital Mülheim, Kaiserstraße 50, 45468 Mülheim, Germany

**Keywords:** Cardiovascular magnetic resonance, Feature tracking, Left ventricular strain, Reproducibility, Sample size, Porcine model

## Abstract

Cardiovascular magnetic resonance feature tracking (CMR-FT) is a novel technique for non-invasive assessment of myocardial motion and deformation. Although CMR-FT is standardized in humans, literature on comparative analysis from animal models is scarce. In this study, we measured the reproducibility of global strain under various inotropic states and the sample size needed to test its relative changes in pigs. Ten anesthetized healthy Landrace pigs were investigated. After baseline (BL), two further steps were performed: (I) dobutamine-induced hyper-contractility (Dob) and (II) verapamil-induced hypocontractility (Ver). Global longitudinal (GLS), circumferential (GCS) and radial strain (GRS) were assessed. This study shows a good to excellent inter- and intra-observer reproducibility of CMR-FT in pigs under various inotropic states. The highest inter-observer reproducibility was observed for GLS at both BL (ICC 0.88) and Ver (ICC 0.79). According to the sample size calculation for GLS, a small number of animals could be used for future trials.

## Introduction

Myocardial strain has been demonstrated as an effective method for the assessment of the regional myocardial function and deformation, and in particular, the cardiovascular magnetic resonance (CMR) tissue tracking approach has been established as a technique comparable to the highly validated speckle tracking echocardiography [19]. CMR feature tracking (CMR-FT) is a relatively novel technique that focuses on endocardial and epicardial contouring and is able to detect the contrast between myocardium and blood pool [4, 14]. CMR-FT has been validated against myocardial tagging technique for the assessment of regional myocardial motion in humans [8, 12, 17]. As every new technique CMR-FT has been widely tested for reproducibility, and what has been already shown in human is the excellent inter- and intra-observer reproducibility, for different parameters and at different field strength MRI scanners [13, 20, 21]. However, since CMR is becoming widely utilized in animal research, there is a lack of standardization, a lack of reference databases and a lack of reproducibility studies. For this reason, a previous study from our group demonstrated the high reproducibility of strain measurements through feature tracking in a model of small animals (mice) which has already been acknowledged in the recent guidelines for animal research [9, 10]. Nonetheless, the main limitation of this previous study was indeed the model, recognized to be not translational enough for a comparison with the humans, in particular regarding the assessment of myocardial function. Large animals, such as Landrace pigs, are instead more suited to investigate myocardial function under various pharmacological interventions given a cardiac anatomy and physiology closer to humans. There is only one study in the literature that has assessed the reproducibility of myocardial deformation parameters in large animals (macaque) [15] and no studies have performed such an analysis in pigs. Accordingly, we performed this preliminary study to evaluate inter- and intra-observer reproducibility of CMR-FT derived strain measurements in a porcine model of pharmacologically induced hyper- and hypo-contractility. Furthermore, we performed a sample size calculation based on global strain values useful to define the number of animals required for future studies.

## Methods

Data from ten landrace pigs were selected from an ongoing experiment at our center. The experimental protocols were approved by the local bioethics committee of Berlin, Germany (G0138/17), and conform to the “European Convention for the Protection of Vertebrate Animals used for Experimental and other Scientific Purposes” (Council of Europe No 123, Strasbourg 1985).

### Experimental setup

**Fig. 1:**
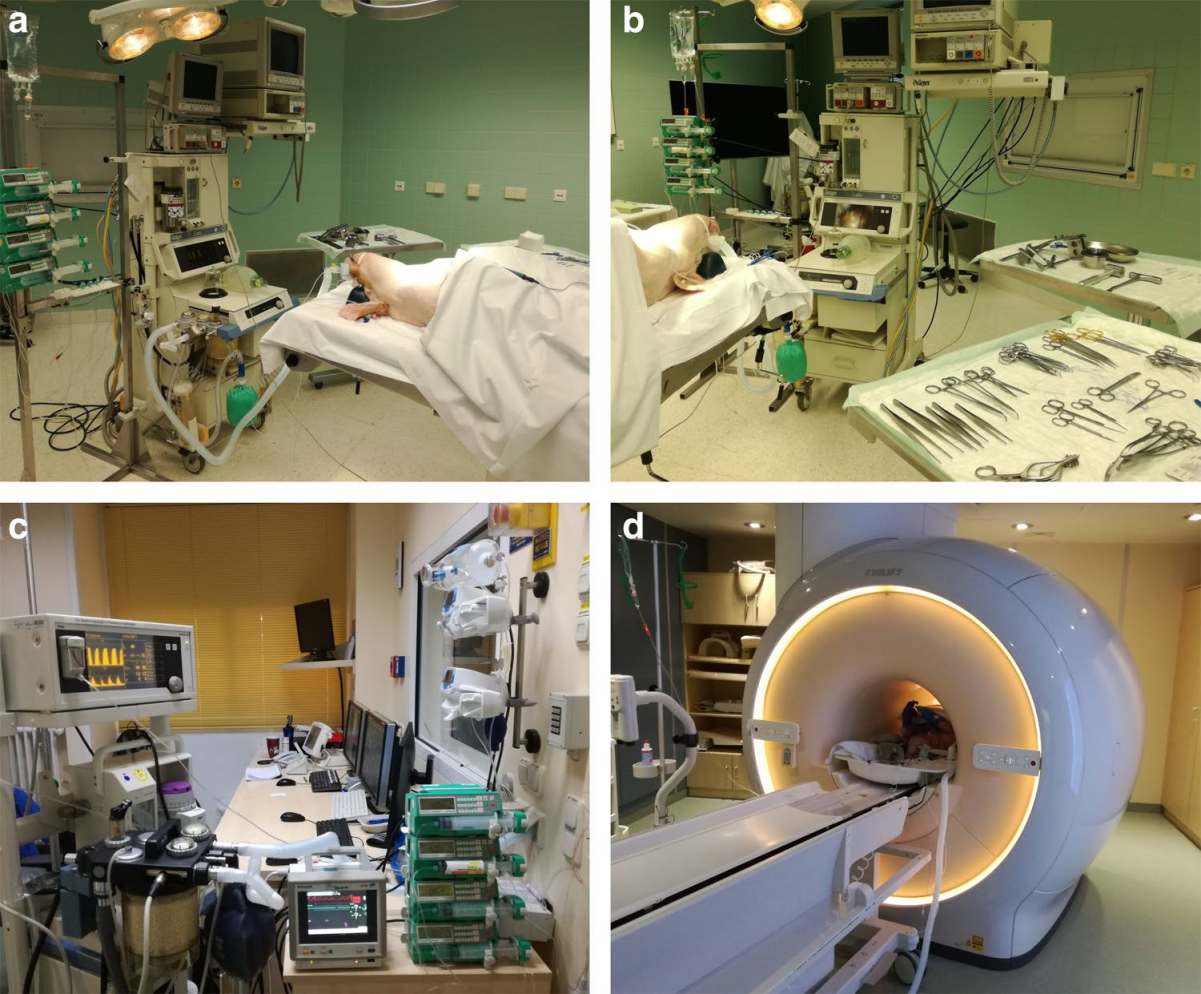
The experimental setting. The animals were acutely instrumented closed chest in the operating room (**a**, **b**) and then transported to the MRI facility where anesthesia and monitoring was maintained during the whole experimental protocol (**c**, **d**)

Briefly, female Landrace pigs (n = 10, 51 ± 10 kg) were fasted overnight with free access to water, sedated and intubated on the day of the experiment. Anesthesia was continued with isoflurane, fentanyl, midazolam, ketamine and pancuronium. Pigs were ventilated (Cato, Dräger Medical, Germany) with a FiO2 of 0.5, an I: E-ratio of 1:1.5, the positive end-expiratory pressure was set at 5 mmHg and a tidal volume (VT) of 10 ml kg^−1^. The respiratory rate was adjusted constantly to maintain an end-expiratory carbon dioxide partial pressure between 35 and 45 mmHg. Under fluoroscopic guidance all animals were instrumented with a floating balloon catheter in the right atrium as well as in the coronary sinus (Arrow Balloon Wedge-Pressure Catheters, Teleflex Inc USA). In order to avoid MRI artefacts, the balloon-tip was cut before introducing the catheters in the vessel. Respiratory gases (PM 8050 MRI, Dräger Medical, Germany), heart rate and arterial blood pressure continuously monitored. Body temperature was monitored by a sublingual thermometer and was maintained at 38 °C during CMR imaging via air ventilation and/or infusion of cold saline solution. The experimental setup can be visualized in Fig. 1a, b.

### Experimental protocols

After acute instrumentation the animals were transported to the MRI facility for measurements, pigs were ventilated with an MRI compatible machine (Titus, Dräger Medical, Germany) (see Fig. 1c, d). After baseline measurements (BL), two steps were performed: (I) dobutamine-induced hypercontractility (Dob) and (II) verapamil-induced hypocontractility (Ver). At each protocol, MRI images were acquired at short axis (SAX), two chambers (2Ch), three chambers (3Ch) and four chambers (4Ch) views. After the MRI measurements were concluded the animals were transported back to the operating room for sacrifice.

### Cardiac magnetic resonance

All CMR images were acquired in a supine position using a 3 Tesla (3 T) (Achieva, Philips Healthcare, Best, The Netherlands) MRI scanner with an anterior- and the built-in posterior coil element, where up to 30 coil elements were employed, depending on the respective anatomy. All animals were scanned using identical comprehensive imaging protocol. The study protocol included initial scouts to determine cardiac imaging planes. Cine images were acquired using ECG-gated balanced steady state free precession (bSSFP) sequence in three left ventricular (LV) long-axis (two-chamber, three-chamber and four-chamber) planes. The ventricular two-chamber and four-chamber planes were used to plan stack of short-axis slices covering entire LV. The following imaging parameters were used: repetition time (TR) = 2.9 ms, echo time (TE) = 1.45 ms, flip angle = 45°, voxel size = 1.9 × 1.9 × 8.0 mm3 and 40 phases per cardiac cycle.

### Image analysis

All images were analyzed offline using commercially available software (Medis Suite, version 3.1, Leiden, The Netherlands) in accordance to recent consensus document for quantification of LV function using CMR. In the strain analysis were included 2Ch, 3Ch and 4Ch cine images, and respectively, three preselected mid-ventricle slices from the LV short-axis stack. The endocardial and epicardial contours drawn on cine images with QMass version 8.1 were transferred to QStrain RE version 2.0, where after the application of tissue tracking algorithm endocardial and epicardial borders were detected throughout all the cardiac cycle. These long-axis cine images were further used to compute global myocardial longitudinal (GLS) strain and short-axis images were used to compute circumferential (GCS) and radial (GRS) strain and strain-rate. The global values were obtained through averaging the values according to an AHA 17 segments model, apex being excluded, as follows: GCS and GRS from averaging CS and RS for 6 basal, 6 mid and 4 apical segmental individual values; GLS from 2Ch, 3Ch and 4Ch averaging 6 basal, 6 mid and 4 apical segments using a bull-eye view.

### Statistical analysis

**Fig. 2:**
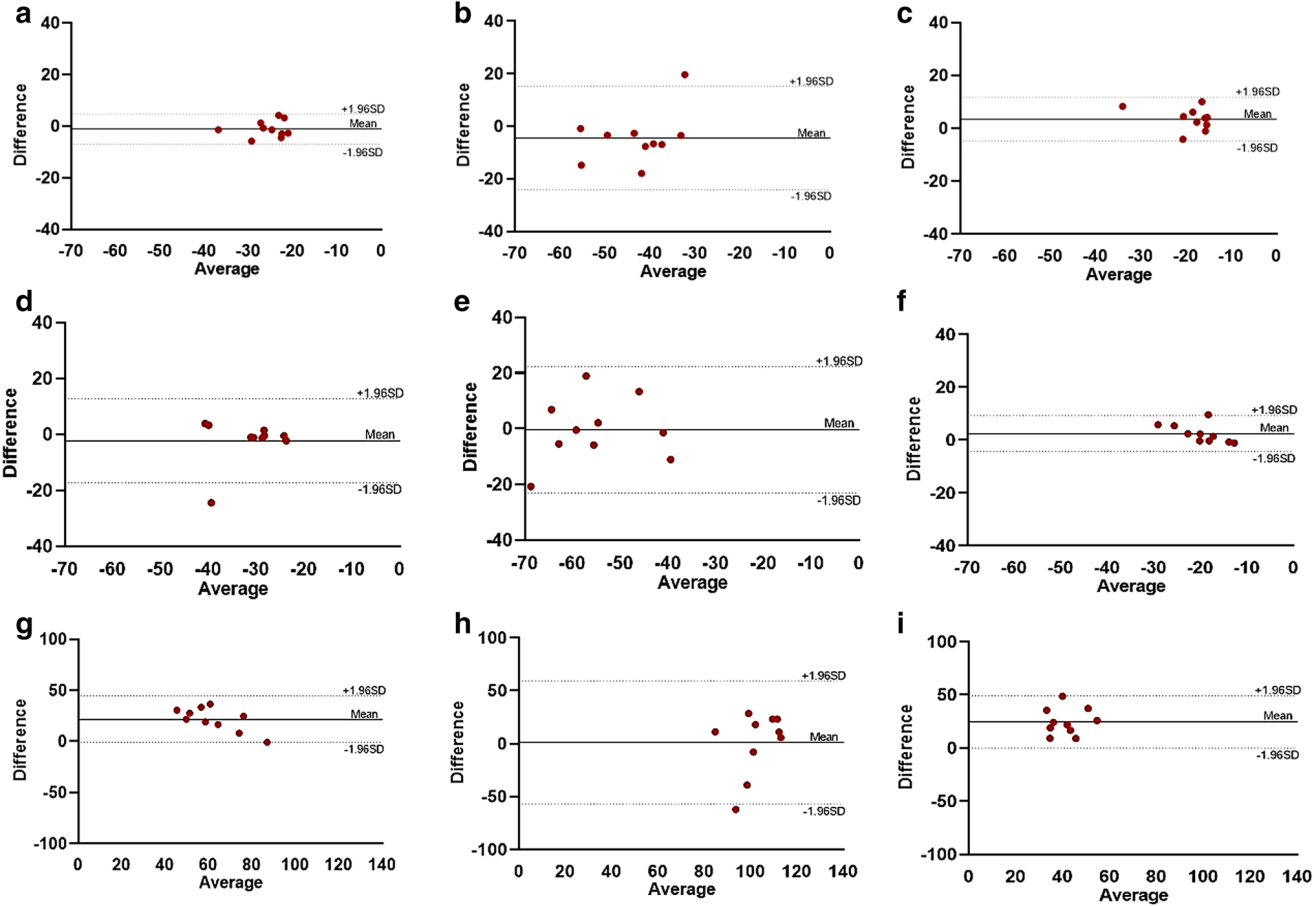
Bland–Altman plots for inter-observer reproducibility of global strain values. Bland–Altman plots showing inter-observer reproducibility for GLS (top row, panels **a**–**c**), GCS (middle row, panels **d**–**f**) and GRS analysis (bottom row, panels **g**–**i**) during BL, Dob and Ver steps respectively. *BL* baseline, *Dob* Dobutamine, *Ver* Verapamil, *GLS* global longitudinal strain, *GCS* global circumferential strain, *GRS* global radial strain

**Fig. 3:**
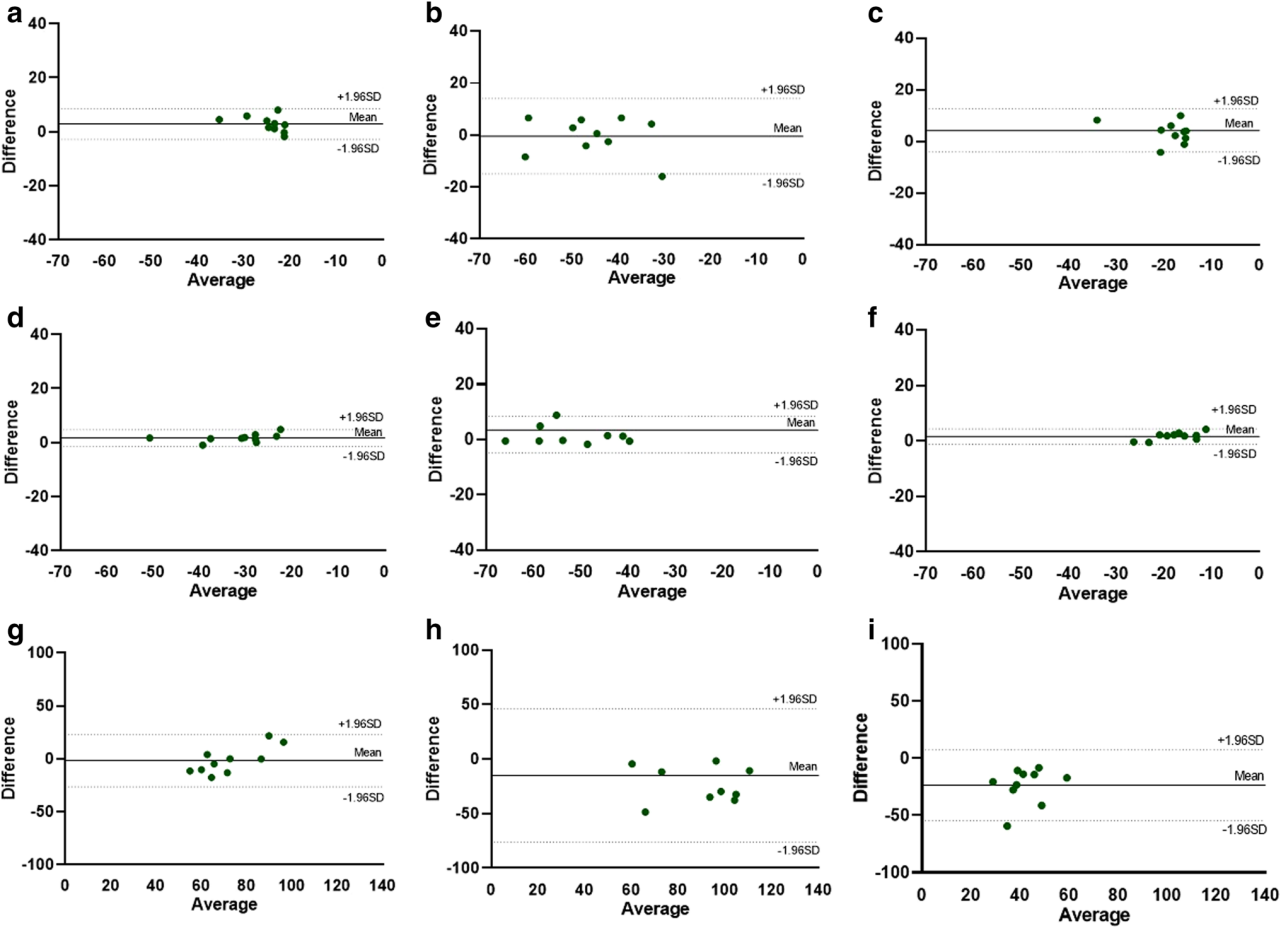
Bland–Altman plots for intra-observer reproducibility of global strain values. Bland–Altman plots showing intra-observer reproducibility for GLS (top row, panels **a**–**c**), GCS (middle row, panels **d**–**f**) and GRS analysis (bottom row, panels **g**–**i**) during BL, Dob and Ver steps respectively. *BL* baseline, *Dob* Dobutamine, *Ver* Verapamil, *GLS* global longitudinal strain, *GCS* global circumferential strain, *GRS* global radial strain

**Fig. 4:**
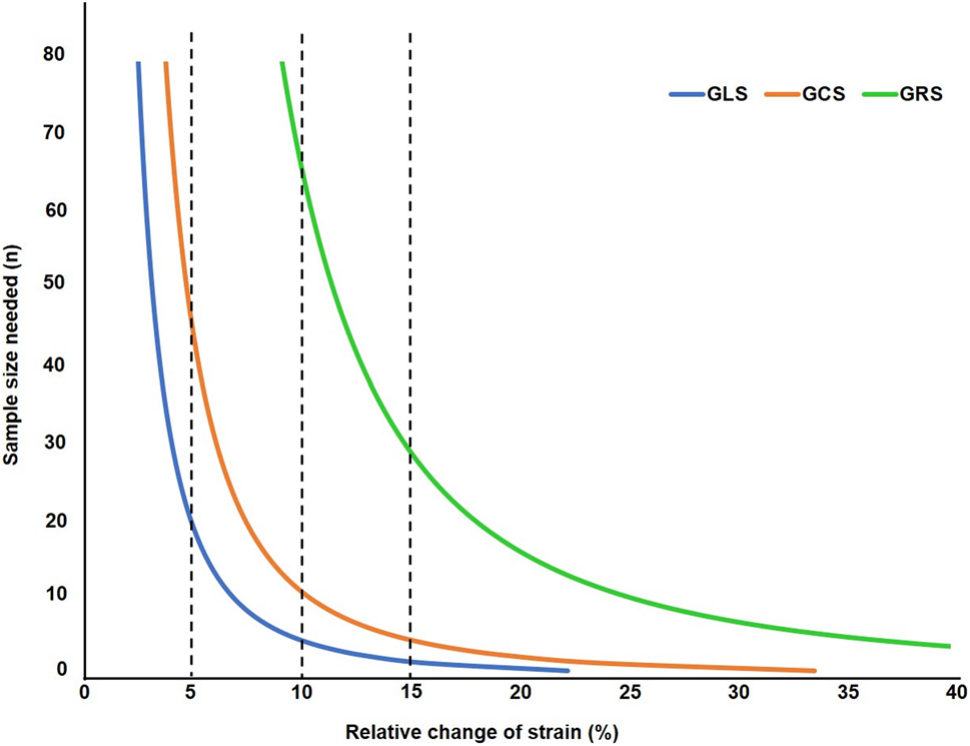
Graphical representation of the sample size calculation for global strain values. Representation of the sample size calculation for GLS (blue), GCS (red) and GRS (green) baseline measurements to detect the desired % relative change of strain with 80% power and α error of 0.05. Dashed lines represent the relative change of strain at 5%, 10% and 15% respectively. *GLS* global longitudinal strain, *GCS* global circumferential strain, *GRS* global radial strain

Data were analyzed using Microsoft Excel and IBM SPSS Statistics version 23.0 software (SPSS Inc., Chicago, IL, USA) for Windows. Figures 1, 2, 3, 4 were made with Microsoft PowerPoint version 17, while Figs. 2, 3 were made with GraphPad Prism version 8. All data are presented as mean ± SD. Data between groups at different inotropic states were analyzed by one-way ANOVA for repeated measurements. Post-hoc testing was performed by Tukey’s test. A p-value < 0.05 was considered significant.

### Reproducibility testing

Data are expressed as mean ± standard deviation (SD). The Shapiro–Wilk test was used to determine whether the data were normally distributed. Nonparametric variables were compared using the Wilcoxon test. A p-value of < 0.05 was considered statistically significant. Inter- and intra-observer reproducibility was quantified using intra-class correlation coefficient (ICC) and Bland–Altman analysis (13). Agreement was considered excellent for ICC > 0.74, good for ICC 0.60–0.74, fair for ICC 0.40–0.59, and poor for ICC < 0.40 (14). To assess intra-observer agreement data analysis was repeated after 4 weeks. All the operators took the measurements twice and the average values were taken.

### Sample size calculation

Study sample size required to detect a relative 5, 8 and 10% change in strain with power of 80% and significance of 5% was calculated as follows (15):

$${\text{n}}\, = \,{\text{f}}\left( {\alpha ,\,{\text{P}}} \right)\sigma 2/\delta$$

where n is the sample size, α the significance level, P the study power required and f the value of the factor for different values of α and P (f = 10.5 for α = 0.05 and p = 0.080), with σ the standard deviation of differences in measurements between two studies and δ the desired difference to be detected. Sample size calculation was performed for baseline values only.

## Results

The volumetric and functional parameters of study population are summarized in Table 1. All studies were completed, and image quality was sufficient to perform CMR-FT analysis. Table 2 demonstrates CMR-FT derived strain parameters obtained by two independent investigators.

**Table 1:** Volumetric and functional characteristics of study subjects

Parameter	Value	
Study population		10
LV EDV (ml)	BL	101 ± 24
	Dob	83 ± 21*
	Ver	108 ± 25^†^
LV ESV (ml)	BL	41 ± 10
	Dob	19 ± 7*
	Ver	66 ± 18*^†^
LV SV (ml)	BL	60 ± 19
	Dob	64 ± 18
	Ver	42 ± 12*^†^
LV EF (%)	BL	59 ± 8
	Dob	77 ± 7*
	Ver	39 ± 9*^†^
Cardiac output (l/min)	BL	6 ± 1
	Dob	9 ± 2*
	Ver	4 ± 1*^†^
Heart rate (bpm)	BL	106 ± 15
	Dob	146 ± 12*
	Ver	98 ± 19^†^
LV Mass (g)		81 ± 20

Results are reported as mean ± standard deviation

*BL* baseline, *Dob* Dobutamine, *EDV* end-diastolic volume, *EF* ejection fraction, *ESV* end-systolic volume, *LV* left ventricle/ventricular

*SV* stroke volume, *Ver* Verapamil

*p-value < 0.05 versus BL

^†^p-value < 0.05 versus Dob

**Table 2:** Comparison of CMR-FT derived average of global strain parameters obtained by observers in ten pigs during BL, Dob and Ver steps

Measurements obtained by two observers (inter-observer level)		
	First observer	Second observer
BL (%)		
GLS	− 26.1 ± 5	− 25.1 ± 4
GCS	− 32.7 ± 8	− 30.4 ± 6
GRS	73.3 ± 9	51.5 ± 17
Dob (%)		
GLS	− 45.1 ± 11*	− 40.6 ± 7*
GCS	− 55.1 ± 12	− 54.7 ± 10*
GRS	103.0 ± 20	101.8 ± 14*
Ver (%)		
GLS	− 20.8 ± 6^†^	− 17.3 ± 5*^†^
GCS	− 18.6 ± 4^†^	− 21.0 ± 6*^†^
GRS	53.9 ± 10^†^	29.1 ± 9*^†^

Measurements obtained by one observer (intra-observer level)		
	First measurement	Second measurement
BL (%)		
GLS	− 26.1 ± 5	− 23.3 ± 4
GCS	− 32.7 ± 8	− 31.0 ± 8
GRS	73.3 ± 9	71.7 ± 19
Dob (%)		
GLS	− 45.1 ± 11 *	− 45.6 ± 9*
GCS	− 55.1 ± 12	− 53.3 ± 11*
GRS	103.0 ± 20	87.9 ± 36
Ver (%)		
GLS	− 20.8 ± 6^†^	− 16.5 ± 3*^†^
GCS	− 18.6 ± 4^†^	− 16.9 ± 5*^†^
GRS	53.9 ± 10^†^	30.3 ± 13*^†^

All the operators took the measurements twice and the average values were taken

*BL* baseline, *Dob* Dobutamine, *GCS* global circumferential strain, *GLS* global longitudinal strain, *GRS* global radial strain, *Ver* Verapamil

*p-value < 0.05 versus BL

^†^p value < 0.05 versus Dob

### Inter-observer and intra-observer reproducibility

Mean differences ± SD, limits of agreement and ICC for strain parameters are given in Table 3. There was an excellent inter-observer reproducibility for GLS during BL and Ver steps, while during Dob the observed reproducibility was good. Regarding the GCS analysis, there was a good reproducibility during BL and Ver steps; while during Dob, the reproducibility was only fair. The GRS analysis showed, instead, an excellent reproducibility for BL, while during Dob and Ver steps the reproducibility was poor. Concerning the intra-observer analysis, the level of reproducibility was generally excellent for most of the measurements. GLS and GCS showed an excellent reproducibility for all steps, while GRS showed an excellent reproducibility during BL, a good one during Dob and a poor one during Ver. Bland–Altman plots demonstrate inter-observer and intra-observer reproducibility for GLS, GCS and GRS analysis during BL, Dob and Ver steps (see Figs. 2, 3).

**Table 3:** Inter-observer and intra-observer reproducibility for GLS, GCS and GRS

	Parameter	Steps	Mean difference ± SD	Limits of agreement	ICC (95% CI)
Inter-observer variability	GLS	BL	− 1.0 ± 3.0	− 6.9 to 4.8	0.88 (0.57–0.97)
		Dob	− 4.5 ± 10.0	− 24.1 to 15.1	0.60 (− 0.35 to 0.89)
		Ver	3.5 ± 4.2	− 4.7 to 11.7	0.79 (0.10–0.95)
	GCS	BL	− 2.2 ± 7.6	− 17.2 to 12.6	0.66 (− 0.21 to 0.92)
		Dob	− 0.4 ± 11.5	− 23.0 to 22.2	0.51 (− 0.23 to 0.87)
		Ver	2.4 ± 3.4	− 4.3 to 9.2	0.61 (− 0.40 to 0.90)
	GRS	BL	21.7 ± 11.6	− 0.9 to 44.5	0.80 (0.21–0.95)
		Dob	1.2 ± 29.6	− 56.9 to 59.3	− 1.60 (− 9.47 to 0.35)
		Ver	24.7 ± 12.6	0.1 to 49.4	0.24 (− 2.03 to 0.81)
Intra-observer variability	GLS	BL	2.8 ± 2.9	− 2.8 to 8.5	0.81 (0.41–0.95)
		Dob	− 0.4 ± 7.3	− 14.8 to 14.0	0.87 (0.45–0.96)
		Ver	4.3 ± 4.3	− 4.1 to 12.7	0.75 (0.01–0.94)
	GCS	BL	1.7 ± 1.5	− 1.3 to 4.7	0.98 (0.77–0.99)
		Dob	1.8 ± 3.4	− 4.8 to 8.4	0.97 (0.89–0.99)
		Ver	1.6 ± 1.4	− 1.1 to 4.4	0.95 (0.36–0.99)
	GRS	BL	− 1.5 ± 12.7	− 26.4 to 23.3	0.79 (0.15–0.94)
		Dob	− 15.1 ± 31.1	− 76.0 to 45.8	0.62 (− 0.50 to 0.90)
		Ver	− 23.6 ± 15.7	− 54.4 to 7.2	0.14 (− 2.43 to 0.78)

Results are reported as mean ± standard deviation

*BL* baseline, *CI* confidence interval, *Dob* Dobutamine, *GCS* global circumferential strain, *GLS* global longitudinal strain, *GRS* global radial strain, *ICC* intra-class correlation coefficient, *Ver* Verapamil

### Sample size calculation for baseline values

The change in reproducibility has an impact on the sample size required to detect significant differences in strain parameters. Table 4 lists the required sample sizes for each strain-derived parameter. For example, to show a relative 10% change in GLS in pigs would require five animals (not measures—Fig. 4). In contrast, 20 pigs are required to detect a 5% change in GLS with CMR-FT (power of 80% and α error of 0.05).

**Table 4:** Sample size calculation for GLS, GCS and GRS (baseline measurements) to detect the desired % relative change with 80% power and α error of 0.05

	Mean difference ± SD pooled	Sample size (n)		
		5%	10%	15%
GLS	− 1.1 ± 4.9	20	5	2
GCS	− 2.3 ± 7.4	45	11	5
GRS	21.7 ± 17.9	NA	68	30

The mean difference is calculated from the inter-observer mean difference analysis. The pooled SD has been obtained by applying Cohen formula: SD_pooled_ = √ (SD_1_^2^ + SD_2_^2^)^−1^

*GCS* global circumferential strain, *GLS* global longitudinal strain, *GRS* global radial strain, *SD* standard deviation

## Discussion

While studies analyzing reproducibility of CMR-FT in humans are already present in the literature [3, 16], works on the reproducibility of myocardial deformation parameters of large animal models are, instead, lacking. The current study was designed, therefore, to assess the inter-observer and intra-observer reproducibility of CMR-FT for the analysis of global LV strain in a porcine model of hyper- and hypo-contractility. Here we show a good to excellent inter- and intra-observer reproducibility of CMR-FT technique in pigs under different inotropic states. Furthermore, sample size calculation demonstrates that for GLS analysis a small number of animals could be enough for future trials. A previous study from our group has demonstrated a high reproducibility in the LV strain measurements in a murine model [9]. This current study provides a more extensive analysis in pigs and confirms the previous one regarding the most reproducible parameters derived from CMR-FT. Good to excellent inter-observer reproducibility was found for global longitudinal and global circumferential strain, whereas radial strain confirms, instead, to be highly variable between repeated measurements, in particular when considering the inter-observers measurements. The weak reproducibility of radial strain has also been reported in previous studies [2, 16, 23, 28]. While global longitudinal strain was the most reproducible parameter during the inter-observer analysis, the intra-observer reproducibility was predictably higher for most of the strain values and excellent for global circumferential strain, as already described in previous studies [13, 22]. In a previous study from our group, we were able to show the positive additional role of LV strain analysis during dobutamine stress in a group of patients with coronary artery disease [18]. A good reproducibility and a low inter-observer variability of dobutamine stress Echo and CMR has been previously observed in human [21, 26] and animal studies [15]. However, only few studies concentrated on the reproducibility of LV strain in hyper- and hypo-contractility model. With this study, we were able to assess the reproducibility of the LV strain measurements under various inotropic states. During the infusion of dobutamine, aiming at an increase of at least 25% of the baseline HR, we were able to observe a clinically significant increase in LV EF and LV cardiac output. In our study, the high HR obtained during dobutamine infusion (mean 146 ± 12 bpm) was, however, detrimental to the reproducibility of the measurements when measured by another observer. This can be explained by a worse resolution of the MRI processed images and by an increase in frame rates under such fast heart beats, as already described in other studies [7, 15]. In one of the studies by Schuster et al. a high reproducibility of CMR-FT in a group of ischemic cardiomyopathy patients after dobutamine infusion for stress test was observed [21]. Nevertheless, it is worth to mention that in that study no reference to the heart rate at which the sequences were recorded was mentioned, making the comparison with our model not consistent. In our study, we were able to show that at lower heart rates, such as during baseline state and verapamil infusion, the reproducibility was generally higher for all the strain values. With the advent, development and availability of computers, large datasets can be used in statistical analysis to calculate the sample sizes necessary for clinical studies. Sample size calculation is an important aspect of study design and enables determination of how large the study sample should be. Estimates of required sample size depend on the variability of the population—the greater the variability, the larger the required sample size. This is particularly relevant for CMR studies, where the role of sample size is extremely useful to test the reliability of new imaging techniques [11, 16]. The same should be applicable to animal studies, where the reduction of the numbers of animals used is of extreme value [25]. In some cases, by using previously published studies, the use of animals can be totally avoided by eliminating unnecessary replications [1]. Modern imaging techniques in conjunction with new statistical analysis methods also allow reductions in the numbers of animals used, for example, by providing greater information per animal [27]. Too small sample size can miss the real effect, whereas too large sample size leads to unnecessary waste of time and resources (animals) [1, 25, 27]. In the already published pilot study on sample size calculation and variability in small animals by our study group, we demonstrated that the number of animals needed to test a hypothesis could be reduced if the effect of animal-to-animal variation on the measurement is eliminated or highly reduced [9]. With the present study, we were able to show that in this cohort a relatively small sample size of animals (not measures) is required to detect a 5, 10 and 15% change in strain parameters for global longitudinal strain. We also observed that a higher sample size is necessary for circumferential strain, and particularly high for radial strain. The ability to apply human‐like settings to model animals increases the chances of translation of new effective diagnostic and therapeutic interventions [24]. The importance of large animal research in the field of human diseases is evident in most medical settings, however, this holds particularly true for cardiology where in terms of anatomy, physiology and size, large animals such as pigs represent the closest comparison to humans [24]. In the European Union, the Directive 2010/63/EU voted in 2010 has been implemented in 2013 in the European Medicines Agency (EMA) guidelines, resulting in restrictions in the use of nonhuman primates in biomedical research (EMA 2014) and promoting instead the utilization of non-rodents species such as pigs and sheep that should be chosen based on their similarity to humans with regard to in vitro metabolic profile [6]. In order to appropriately assure translational success and safety, at least two animal species that are phylogenetically somewhat apart like a rodent and a non-rodent species are necessary. Without necessarily requiring closeness to man, this is the general rule supported by the current international guidelines like the International Council for Harmonization of Technical Requirements for Pharmaceuticals for Human Use, Guidance M3 (Revision 2; 2009) [6]. Nonetheless, the employment of large animal models carries ethical problems and higher costs, mainly because of the size of the animals and husbandry needed when compared to smaller models [24]. It is evident that all the necessary methods should be introduced to reduce, refine and replace the unnecessary animal experiments [5]. In accordance with the 3Rs principles on animal use (Directive 2010/63/EU), a scientifically satisfactory method or testing strategy, not entailing the use of live animals, should be used wherever possible [6]. For this reason, our preliminary study could be paving the road to the realization of an open access database of cardiovascular magnetic resonance data that could be of great need for future laboratory experiments, to reduce the number of animal experiments performed and to be utilized as a platform for simulation and testing of novel compounds. The experiments were performed during anesthesia, being a possible confounder for reproducibility of the measurements. The animals were not awake limiting the translation to clinical settings. The study is limited due to the small number of animals and larger sample size may be required to detect more subtle differences. The addition of a 25% dropout rate (proportion of eligible subjects who will not complete the study or provide only partial information) before planning a study will further increase the final sample size.

## Conclusion

Global LV strain parameters analyzed by CMR-FT analyzed in a large animal model (pig) of hyper- and hypo-contractility are highly reproducible. The most reproducible measures are global circumferential and global longitudinal strain, whereas reproducibility of radial strain is weak. Sample size calculation are an essential tool that could help to reduce the number of animal experiments and databases on large animals can be used as a platform to test the effect of novel compounds.
